# Factors which influence social connection among cancer caregivers: an exploratory, interview study

**DOI:** 10.1007/s00520-024-09095-w

**Published:** 2025-01-14

**Authors:** Eva Y. N. Yuen, Shadow Toke, Helen Macpherson, Carlene Wilson

**Affiliations:** 1 School of Nursing and Midwifery, Faculty of Health, Deakin University, Burwood, VIC Australia; 2 Centre for Quality and Patient Safety-Monash Health Partnership, Institute for Health Transformation, Deakin University, Burwood, VIC Australia; 3 Monash Health, Clayton, VIC Australia; 4 School of Exercise and Nutrition Sciences, Deakin University, Burwood, VIC Australia; 5 Centre for Epidemiology and Biostatistics, University of Melbourne, Parkville, VIC Australia

**Keywords:** Cancer, Caregivers, Qualitative, Social support, Emotional support, Social connectedness

## Abstract

**Background/aims:**

Social connectedness is increasingly recognised as influencing health outcomes in cancer caregivers; however, there is little understanding of factors which foster feelings of social connectedness among caregivers when providing care. We sought to examine from the caregivers’ perspective, factors which contribute to perceived social connection when providing care to someone with cancer.

**Methods:**

Semi-structured interviews were conducted with 20 caregivers of people with cancer. Participants were recruited through social media and were eligible if they were aged 18 + years and had provided care to someone with cancer in the preceding 3 years. Data were analysed thematically using NVivo.

**Results:**

Following thematic analysis of interview data, six overarching themes emerged detailing caregivers’ experiences with social support, networks, and connectedness. Themes included: people in caregivers’ social networks and communication frequency, supportive communication with people in social networks, challenges with talking with others and seeking support, receiving instrumental support from social networks, impact of the carer role on friendships and community activities, and factors which fostered perceived connectedness.

**Conclusions:**

For caregivers of people with cancer, our qualitative findings suggest the importance of receiving emotional and instrumental support from social networks to cope with, and alleviate the stress and strain of providing care. Development and empirical testing of strategies and interventions that improve social support seeking and subsequently perceived connectedness among caregivers are recommended to improve health and wellbeing.

## Perceived social connection and its relationship to wellbeing among cancer carers: an exploratory, interview study

Family and friends often play an important role in providing support and care to people diagnosed with cancer [1]. Although providing care can be rewarding and foster stronger relationships between the caregiver and care recipient, it can also be stressful and burdensome, and result in negative health outcomes for the caregiver [2]. Studies have shown the importance of social support on health outcomes of cancer caregivers, by enabling improved coping through informational, instrumental, and emotional support [3]. Greater social support among caregivers has been associated with improved quality of life [4], reduced caregiver burden, and lower depression and anxiety [5, 6]. However, taking on the caregiving role and associated responsibilities is often sudden and unexpected, impacting the quality, availability, and frequency of interactions with others. Barriers to caregivers engaging with others include lack of time and energy, perceived emotional burden of talking to others, and not wanting to socialise with others without the care recipient [7, 8].

Evidence also points to the impact of social connectedness on caregiver health and psychological outcomes. Social connectedness is described as perceptions of close, meaningful, and constructive interpersonal relationships with other individuals, groups, or the community [9, 10]. Although individuals may report having adequate social support (described as the quantity and quality of available instrumental, emotional, information and/or appraisal support from social networks [11]), they may still report feeling lonely, or lacking a perceived sense of belonging, or social connection with their networks [12]. Among cancer caregivers, both social support and social connectedness were found to protect against caregiver burden [13]. However, only social connectedness, and not social support, buffered against the negative effects of psychological symptoms [14, 15]. These findings suggest that available social support networks are likely to assist caregivers with the day-to-day responsibilities of providing care and can alleviate burden associated with the caregiving role. However, availability of social support is insufficient to protect against the psychological sequalae of caregiving; rather, findings suggest the importance of fostering perceived connectedness to enhance psychological health and wellbeing.

Although there is recognition of the need to promote social connectedness in caregiver populations [16], to date, there is little understanding of factors that foster feelings of social connectedness among caregivers when providing care. Understanding factors that contribute to perceived social support and connection in caregivers has the potential to inform the development of strategies to improve their overall health. The current study sought to understand influences on caregivers’ perceived social connection while providing supportive cancer care.

## Methods

A qualitative, semi-structured interview study was conducted with caregivers who were currently providing, or had provided, unpaid care to someone diagnosed with cancer in the preceding 3 years, and who were aged 18 + years, and residing in Australia. Ethics approval was obtained from the Monash Health Human Research Ethics Committee (RES-21–0000-250A). Research was conducted in accordance with the Australian code for the responsible conduct of research. Participants received a $30 Coles eGift voucher as a token of thanks for their time.

### Data collection

Participants were recruited through social media channels (e.g., Facebook). Interested individuals were invited to complete a pre-screening survey to express their interest in participation and determine their eligibility. The pre-screening survey included the Social Connectedness Scale [10], an 8-item measure designed to assess perceived connection and belongingness. Items were rated on a 6-point Likert scale (strongly disagree–strongly agree). Total score ranged from 6 to 48 with higher scores suggesting higher perceived social connectedness.

Only eligible individuals were contacted via telephone by the research team to determine their interest in participation, and to organise a time for the interview via Zoom or telephone. Verbal consent was obtained at the commencement of interviews. A semi-structured interview guide was used, and participants were asked to share their experiences interacting with people in their networks while providing supportive cancer care. Interviews were conducted by a female researcher with a biomedicine background who had training and experience conducting interviews with people from culturally and linguistically diverse communities. Interviews were conducted between June and September 2023, and audio recorded.

Regarding sample size, a pragmatic approach was adopted, with the aim to recruit a minimum of 20 participants to achieve data saturation. A sample size between 20 and 30 has been shown to be sufficient to reach theoretical saturation in studies using grounded theory approaches [17].

### Data analysis

Social connectedness in participants was categorised by tertile as low (8–22), moderate (23–35), or high (36–48) scores. Interview transcriptions were conducted using Otter.ai. Participant details were removed from all transcriptions, and recordings were removed from Otter.ai following transcription to maintain participants’ privacy. Data were analysed using Braun and Clarke’s six-phase approach to thematic analysis [18]. This approach emphasises immersion and familiarisation with the data prior to formal coding. Interview responses were then coded in a systematic manner for all interviews. An inductive, “data driven” approach was used to code [18] data, and an interactive and recursive approach was used to categorise data into themes and sub-themes to understand and capture participant experiences. All data were coded and thematically grouped by the female PhD-educated lead author (EY) with experience in cancer caregiver supportive care research and conducting mixed methods studies. Resultant codes and themes were discussed, reviewed, refined, and confirmed with research team members (ST, CW).

## Results

Twenty caregivers participated in the interviews (see Table 1). Most caregivers were providing care to their partner (*n* = 11; 55%) or parent (*n* = 6; 30%), and the majority were female (*n* = 15; 75%). Care recipients varied in their cancer diagnoses; however, half the sample were diagnosed with the most common cancers (breast, colorectal, prostate, lung, or melanoma; *n* = 10). Social connectedness scores varied across the sample; eight caregivers reported low scores, six had moderate scores, and six had high scores. Five overarching themes distinguished the impact of social connectedness: people in the caregivers’ networks and how often they communicated; supportive communication exchanges with people in the social network; challenges and barriers when talking to others and seeking support; receiving practical support from social networks; and impact of the caring role on friendships and community activities (see Table 2 for supporting quotes).

**Table 1:** Participant characteristics

Participant ID	Participant gender	Social connectedness score	Care recipient is my:	Care recipient cancer type
CG001	Male	20	Parent	Breast
CG002	Male	22	Partner	Melanoma
CG003	Female	31	Partner	Head and neck
CG004	Female	41	Parent	Melanoma
CG005	Female	44	Partner	Prostate
CG006	Female	25	Partner	Colorectal
CG007	Female	35	Parent	Neuroendocrine
CG008	Female	44	Partner	Oesophageal
CG009	Male	48	Partner	Lung
CG010	Male	18	Parent	Lung
CG011	Female	10	Partner	Brain
CG012	Male	10	Parent	Ovarian
CG013	Female	22	Friend	Lymphoma
CG014	Female	13	Friend	Breast
CG015	Female	29	Partner	Prostate
CG016	Female	40	Partner	Brain
CG017	Female	34	Partner	Colorectal
CG018	Female	47	Partner	Lymphoma
CG019	Female	35	Parent	Oesophageal
CG020	Female	30	Grandparent	Stomach

**Table 2:** Supporting quotes for themes and subthemes

Theme/sub-theme	Quote/s
People in caregivers’ networks and communication frequency	
Who caregivers interacted with in their social networks	“I’ve got my brother-in-law in the UK, he phones once a week, obviously, chatting about how my wife is and how's the week been” (CG09, male caring for partner, SC48) Some [friends] we went through Bible college with…so we’ve known them for over 20 years… and some people in our local community…there’s also just people…we get on well with…like…an old neighbour of ours… or other people in our music group” (CG16, female caring for partner, SC40) “I work with psychologists and counselors. I’ve got that link directly, I suppose they know me in a sense, in a work relationship. They’re really great and are always happy to have a chat and check in on me as well.” (CG19, female caring for parent, SC35)
Availability of, and relationships with, family members	*Participants with moderate to high social connectedness* **“[**Our children] all live reasonably close, like distance wise to each other.” (CG05, female caring for partner, SC44) “I’ve got a close sister as well. She rings. She tells me to ring whenever I need to, rather than her ringing all the time. She knows my time constraints” (CG08, female caring for partner, SC44) “…One of my daughter’s is psychologist…she’s been really good and just has good advice and stuff” (CG15, female caring for partner, SC29) *Participants with low social connectedness* “Noo.. I mean, look, I’ve got a sister here that lives in [City1]. I don’t really have a lot to do with her. She comes here sometimes. And my other sister lives in [State2].” (CG01, male caring for parent, SC20) “I have a brother, who lives about 400 or 500 [kilometres] away. We don’t share an intimate relationship. And my parents are both gone. So, it really it’s, you know, it’s two things. It’s number one where I’ve moved to a regional centre. And secondly, is the fact that my family is aged and past. So, family wise, there’s not a lot of shoulders. Not a lot of help…” (CG02, male caring for partner, SC22) “Well, we haven’t got any [immediate family], I’ve got an aunt and uncle. So, we live down on the [City1], and they’re in [City2], and they have health issues of their own. So, I’ve not really had any support from them…I only have my dad and he’s overseas, and my husband’s family all overseas. And they’re all well, he’s got his sister, but his parents are elderly. So, we haven’t got any real kind of extended family support.” (CG06, female caring for partner, SC25) “I have an older brother, but he so he lives overseas, so it’s in. I mean, in Australia, it’s definitely my mum and my dad. So it’s a bit tricky for him for her and, you know, because she obviously needs to, she needs the support as well.” (CG10, male caring for parent, SC18)
Frequency of communication with social networks	“Mmost of [our children] ring up every day or send me a message and I do with them so it’s all kind of even though they they’ve moved away from home. We’re still close for us”. (CG05, female caring for partner, SC44) “My cousin I speak to her pretty much like every day…I went to Sydney with her three four weeks ago to support her when her daughter went and had surgery and things so we’re super close. And yeah, like my auntie and my Mum, I’d say [I speak to them] probably every second day…definitely got like quite a close family in a sense where you can kind of tell anyone anything”. (CG19, female caring for parent, SC35) “We keep in touch with each other up every second day. So, it’s just, you know, families. And so we always kind of look out for each other and that kind of thing”. (CG05, female caring for partner, SC44) **“**No, there’s some days I don’t talk to anybody. Yeah. Or text anybody. So, friends from running [group] probably maybe three days a week or so. Yeah. Work colleagues, I’m closest to if I haven’t seen them in a meeting then. Maybe once a week, yeah, but no, there's not contact daily with anybody.” (CG06, female caring for partner, SC25)
Supportive communication with people in the social network	
Open and honest conversations about cancer and demands of the caregiving role	“Well, I find it easy now [to talk about their situation]. Didn’t feel kinda (sic) easy at the beginning, because it was very upsetting when you were talking to people about it, you know. As I said, the more you come to terms with it, you know, at least I don’t find it difficult to talk about it now.” (CG09, male caring for partner, SC48) **“…**Talk about…what was happening…what stage you’re at what the doctors were saying…Because you’ve never had anything to do with [cancer] before…so you got to talk to someone, to the I mean, I, that’s how I work things out. I talk to someone to try and get your head around things. And they have a different perspective on something” (CG17, female caring for partner, SC34) **“**Because cancer is just your normal life now…So it doesn’t have to be a big emotional unburdening or something every time you talk about it, but it is just normal. (CG16, female caring for partner, SC40) “…I work with psychologists and counselors..…and they’re trained to be non-judgmental…I just find having a chat with someone that’s kind of separated so it’s not someone that knows all my intricate details…I know that whatever we say, that’s literally where it starts and stops.. [We] definitely [have] quite a close family in a sense where you can kind of tell anyone anything. But… I suppose it’s just that ability to speak to an outsider, as such where you can just blurt everything out, and you’re not worried about them then worrying about you, that sort of thing. (CG19, female caring for parent, SC35)
People regularly “checking in” to offer support	“Obviously, as I say, when I’m at work, every second week, I’m away at work. And obviously, it’s the first thing when I get to work. People always ask, ‘How’s your wife?’ You know, I was married. So, you know, I do talk to those [colleagues].” CG09, male caring for partner, SC48) There’s a couple of people [at work] that know what’s happening. Just because we’re, I suppose, got a bit more of a personal connection. So yeah, generally the one my team, she [will] raise [it] probably at least once a fortnight just to check again. And more. So does it just as a general, hey, I haven’t spoke (sic) to you for a while. Yeah. What’s happening?” (CG19, female caring for parent, SC35) “Just little things [family members] do as well is sort of by phone ringing or texting me or our children with just a funny, providing a lot of emotional support to our children because I’m probably not as present for that.” (CG04, female caring for parent, SC41) “[Family members will] text me first, you know, on occasion and say, How are you going? How’s things going? You know, do you need anything? Or you know, something along those lines? Yeah.” (CG20, female caring for grandparent, SC30) “So don’t just have to be medical, anything in life. If someone says like, you know, would you like a cup of tea mum, or if you need anything, just let me know. And that’s enough to get through things and cope with things”. (CG05, female caring for partner, SC44) [Two of the care recipient’s friends were] always ringing me up…one fellow even turned up and stayed with me while [the care recipient] was unhealthy, while he was [receiving] palliative care at home. But he come (sic) and stayed with me so I’d have support… he come for that week when we realized that day was going to die.” (CG17, female caring for partner, SC34)
Validation and appraisal from people in their networks	“I think they’re just a bit more understanding. If something does come up, there’ll [say things] like ‘of course, you poor thing’, [or] ‘thinking of you’, whatever it is, or they’ll check in and ask you, ‘how’s your Mom? How’s everything going?’ And like, my husband just obviously gets it because he sees it…So I think it’s just more their attitude…and…they’ll write it…in birthday cards and stuff… ‘you’re so strong, and amazing’ I think, I feel a bit like, validated.” (CG07, female caring for parent, SC35) I think it’s just because [friends will say] ‘yeah, totally get that’. And then they’ll share something back whether it’s similar or what’s happening to them. So I kind of feel just that, I suppose. reassurance that ‘Yeah, been there, done that…same kind of things happening here’. And it’s X, Y, and Z. I feel that that just kind of makes you feel heard. Acknowledged, and that it’s not the end of the line, but like it’s kind of like a normal thing to happen. Or feeling to have. So yeah, I kind of feel that that’s probably that sort of validation, I suppose”. CG07, female caring for parent, SC35)
Understanding from other carers who have “walked that road”	“It’s good [that the friend is also a caregiver], because like, you can sit there and talk to them, and they understand where you’re coming from. Yeah. So having someone that you can sit there and talk to they actually understand that is very helpful…[we provide] emotional support for one another, if one of us is having a particularly bad day, we’ll ring the other one and just vent our hearts out, you know, like, yell out everything, guilt, cancer everything, so to speak. And then yeah, like, it’s just an outlet for us to get our frustrations out” (CG12, male caring for parent, SC10) “I do think it helps [to talk to other people going through a similar situation]. Yeah. Not only does it help them It helps us as well… [it helps] take you away from your own set of problems. And you move on to thinking you know, your problems aren’t as bad as they would be.” (CG08, female caring for partner, SC44) “My best friend in Sydney, she doesn’t have children. She’s not married, hasn’t had that sort of, like cancer or illnesses really around. So, it’s kind of like a, she doesn’t get the stress of sorting your kids out, for example. And the million things you have to think about just to be able to jump on that plane in the first place. So yeah, it’s kind of like, you can tell them, but then you just kind of go, you would never understand that. So yeah, it’s just, I think, yeah, when people have similar situations, you just know that they get what you’re saying…” (CG19, female caring for parent, SC35) “So, whereas with my friends, no one’s kind of been in a similar or same situation. So, it’s kind of like, I feel would have been more beneficial still would be to have that support from people that have that lived experience…I suppose an area of support for me that was lacking was just that people that have walked that road before, to really be able to get what it is that you’re saying.” (CG19, female caring for parent, SC35)
Challenges and barriers when talking to others and seeking emotional support	
Reluctance to burden family members and friends	[I would like more interactions with friends], but they [have] got busy lives, I can’t be a burden to them. That’s normally where people’s families come in. I think as I don’t have that family support that’s what’s that's a big, big missing link in my life.” (CG06, female caring for partner, SC25) “I think friends, friends are different. Because they have their own family and their own issues and its own health issues, things like that. I mean, sure they’re there. But it’s you don’t want to compound their problems with something you’re going through.” (CG11, female caring for partner, SC10) “Or they won’t, but I just don’t see the sense in burdening [our friends]. I mean, it’s a problem shared is a problem halved, as the old saying says, But, you know, I think people have got better things to do, than to sit and listen to me lamenting the problems we have at the moment.” (CG02, male caring for partner, SC22) “So, I’ve only got some friends and colleagues at work that they’re great to talk to and stuff and the friends I have, get out and go for a walk with or go for a run. I’ve been able to chat to them. But again, they have busy lives, and you just don’t want to kind of be the two of how can I say demanding? I don’t want to be…” (CG06, female caring for partner, SC25) “…I had the counselor for a while since [son] died…So when, when things would build up too much [I would talk to them] because a lot of the time, you don't want to burden your own family, or like your daughter with something. CG17; female caring for partner, SC34) “I think [friends are] always there and…more than happy to listen. I guess I it’s more me. It’s more my own thing where I feel like, I feel like I would be burdening I don’t know if burdening is the right word I feel like I would be burdening other people with. I don’t want to dump them with my problem, or with my own issues… [friends have] their own families and they’ve got their own lives to worry about. So, I don’t want to always just keep on ranting about the same stuff. Same things over and over again” (CG10, male caring for parent, SC18)
Others don’t understand their circumstances	“…I think for someone who’s not experienced it…I don’t think they fully understand…sometimes in the conversations, obviously, you, you get a bit frustrated, because you feel they don’t fully appreciate and understand the situation, you know, but, of course, that’s fully understandable. (CG09, male caring for partner, SC48) “…You try and explain that your role’s a permanent role, you try and explain what you’ve got to do. And then [friends make comments like] it can’t be that hard to do that, [or, it can’t] take that much time to do that’. Just comments like that [make it] obvious that they don’t understand that being a live-in caregiver is not something that you can just get up and go out for coffee when you feel like it… In the beginning, it annoyed the absolute hell out of me, it frustrated me to no end. Now, I’ve just kind of got used to it…They don’t comprehend the responsibilities I have… how much work is involved in it… they never really asked whether or not that was because as they weren’t sure if they were allowed to ask…you sit there and try and explain [the caring role] to someone as a non-caregiver and they have no idea, or anything about it, you know, what to do…And when you try and bring it up to them, they feel uncomfortable with it… so it’s not so much of a topic that we actually discussed that much.” (CG12, male caring for Parent, SC10) “But the thing is, not every not everyone understands what cancer is about…It’s just they don’t understand what someone could go through…I think they understand it to a degree but…I don’t think they [understand] what I do… I just feel that… if you’re saying everything is the issue that either the other people switch off or, you know, it’s just like, I’m bitching, so, that’s why I keep most of it to myself.” CG01, male caring for parent, SC20) “And [friends] don’t know how to react. They don’t want to offend you. They don’t know how to respond… (CG11, female caring for partner, SC10) “Sometimes [friends] don’t quite get the level [of responsibilities] … like they’re all very sympathetic and lovely. But I think sometimes just the day to day like the weight of it probably doesn’t register.” (CG07, female caring for parent, SC35)
Lack of trust in others and reliance on oneself	“And though they are there, and which I’m so very grateful for it. Yeah, and I’ve had some extraordinarily wonderful, and I do have some wonderful friends. But it’s yeah, that trusting and opening up to people is quite I find that quite difficult anyway.” (CG11, female caring for partner, SC10) “…[friends] have been here, but it’s a lot of these things are very personal. So you, you know, you generally struggle through them yourself. Yeah.” (CG11, female caring for partner, SC10) “I tend to find it hard to really trust people, particularly people I’m not close to or haven’t had a, or I haven’t had some form of friendship or relationship with for a long period of time. But that’s more based on me, it’s not really related to my situation, or whatever. It’s more just my experiences in my life where, you know, I’ve been let down by people…” (CG10, male caring for parent, SC18) “That’s another personal one. Probably rarely, for me. I don’t trust very [much]. That’s just my personal upbringing. And it’s just my life journey that I don’t trust easily. And yeah, I give my trust very, very rarely… I guess because I don’t have family to rely on.” (CG11, female caring for partner, SC10)
Receiving instrumental support from social networks	
	“…if I couldn’t make an appointment with [the patient]…My mom was really good…She would go appointments with him…because he couldn’t remember things… So that’s where I had to rely on like my mom or my daughter. When I couldn’t make it [to an appointment], one of them would go with him for me so that we could get the real picture.” (CG17, female caring for partner, SC34) “[The neighbours] all say, if you need anything, let me know or you need anything from the shop.…when I fly home [from fly in fly out work] every second Tuesday, they pick me up from the train station and bring me home because they know that my wife can’t.” (CG09, male caring for partner, SC48) “…I have sort of a brother and a sister who do not live locally, but sometimes come and stay and just take the kids out and do some washing or hang out just running our household while we eat. Still keep running our household as well. But with a bit more support that week or for that couple of days, they live about 500 kms away.” (CG04, female caring for Parent, SC41) [My daughter would] take [the patient] some days to chemo…And at the end, because I had him here at palliative care. When he died at home, and she would sit she would come and sit with him and everything. Yeah, she was really good.” (CG17, female caring for partner, SC34) **“**They’ve offered to help with anything…They’ve offered to take [patient] down [to the hospital for treatment]. At this stage, I’ve said, No, that’s fine. I’ll take him down. But they said if it comes to the stage where it’s too much going backwards and forwards… I’ve got assistance with that if I needed.” (CG08, female caring for partner, SC44)
Impact of the carer role on friendships and community activities	
Changes in the perceived value of social interactions: “can’t be bothered going out”	“I’m not particularly bothered by [seeing less of my friends]. Anyway, as I’ve gotten older, I’m not…as social…and my social networks have drilled down as well…the circle’s a lot smaller than what it used to be. And yeah, it doesn’t particularly bother me that I don’t have that capacity to go out and be here and do that. I’ve got something much more important on my plate. So, it pales in comparison.” (CG20, female caring for Grandparent, SC30) “…Now [I interact with others] more on [my] own terms… Whereas before you might have made allowances or you know, I don’t know if it’s called selfish or whatever, but you just don’t want to do the crap, I suppose. You know? Yeah. So you don’t.” (CG17, female caring for partner, SC34)’ “I actually did have a few friends. I used to do a lot of stuff with people. But now I just don’t I just don’t…but I’m happy about that. Because I really can’t be bothered. I think I think you just sort of get your life to something that’s, you’re happy with. I can’t be bothered going out for dinner with people and making small talk. And yeah, of course, I’m not really proud of that life anymore…It’s just a bit too difficult.” (CG15, female caring for partner, SC29)
Lack of time and capacity to develop and maintain friendships and community ties	“Just random people I’ve met that kind of could’ve become friends if they understood the whole caregiving scenario, but they took that as in, I didn’t want to get to know them. So yeah. I think it has caused a few friendships because people don’t understand. As a caregiver, we can’t just drop everything, go and have drinks and all that…I had a whole group of us that used to go out to the archery range. Yeah. Yeah, but since I haven’t really gone out there that much, I’ve kind of lost contact with most of them (CG12, male caring for parent, SC10) “I just don’t have the mental capacity to really invest in friendships. [That is how I] feel by the time I’ve sorted out, work, kids, and then everything else that’s going on and trying to work out…it’s just that sort of mental overload of going. I just don’t need anything else on my plate right now to think about… [there is also] the guilt factor that other people are trying [to maintain the friendship], and I’m not… With social interactions, I don’t want to commit to people to things and then not be able to do them (CG19, female caring for parent, SC35) “I was involved with a Festival of Wearable Arts. I was invited to participate, and I had to say, ‘Look, I just can’t…that was very personal for me, and very difficult that I can’t get back into something that I loved and enjoyed… it’s something that I’ve had to pull away from, and I have no idea when I can get close to that again…other community things like the Historic Society and general public meetings around town, I, yeah, I can’t commit…that's what I say to them. I can’t give you my time to, yeah, I don’t want to let you down” (CG11, female caring for partner, SC10)
Changes to friendships due to the cancer diagnosis	Certainly, I guess a lot of the conversations now is centered around [Patient’s] condition. It’s an interesting time…it’s one where you find fairweather friends and all weather friends…and I guess some people are not comfortable with contacting you… because they don’t know what to say. But no, you find… very quickly, who becomes very reliable. And those who, you know, tend to drift into the background… surprisingly, so at times.” (CG02, male caring for partner, SC22) “But I just think it’s interesting like most people you know, a lot of people just don’t want to [talk to you]… like my best friend…She never calls me… and I get really upset because…I’ve known her since I was two and it’s been 60 years and stuff but she never calls me and I get really upset… she can’t deal with it you know.” (CG15, female caring for partner, SC29)
Factors which fostered perceived belongingness with a community and/or social groups	
	“…the groups that have been part of for a long time and part of with my family, not just on my own. The groups that have a whole range of different types of people. And everybody’s got everybody’s got stuff going on in their lives, but…everybody’s quite supportive of each other.” (CG16, female caring for partner, SC40) “Besides just everyone, everyone just accepts who you are…. No competition or anything because everybody’s friends”. (CG17, female caring for partner, SC34) “[I feel I belong to groups because of] communication and I suppose action, like there have been some friends who have set go at least as about the action. Who might have said, let me know if there’s anything I can do. And often when people make that request, it is very well intention, then quite a genuine request, but it’s very open ended… And that would be where the sense of belonging comes from, I think, communication and responsiveness.” (CG04, female caring for parent, SC41)

### People in caregivers’ networks and communication frequency

#### Who caregivers interacted within their social networks

Caregiver’s social networks included immediate and extended family members (e.g., adult children, siblings, parents, cousins), friends, neighbours, colleagues, and community members (e.g., those living in a small rural town). Some caregivers described interacting with other caregivers in hospital environments. Several identified interacting regularly with colleagues who were psychologists and trained to provide non-judgmental listening as part of their profession, which helped them feel supported.

#### Availability of, and relationships with, family members

Caregivers with moderate to high social connectedness frequently described high family support, and having close, “strong” relationships with family members before the care recipient’s diagnosis of cancer. These caregivers said that family members would often “look out one for one another” and, following the cancer diagnosis, family members would offer more support than previously necessary to assist the caregiver and care recipient. Some caregivers found they became closer to family members who offered emotional and/or instrumental support following the diagnosis.

By contrast, participants with low social connectedness lacked family members they could rely on to provide emotional and/or practical support. Family members either lived overseas, were elderly, or had their own health issues, and thus had limited capacity to assist. One caregiver described physical but not emotional closeness with family, with this precluding asking them for support. Caregivers with low family support reported having to rely on themselves to manage caregiving responsibilities. Additionally, caregivers experiencing low levels of family support indicated dependence on the healthcare system.

#### Frequency of communication with social networks

Frequency of communication with people in their social networks varied with extent of social connectedness. Caregivers with moderate to high social connectedness described frequent communication with family members and friends (daily, or several times per week) via telephone or text messages. Some caregivers with higher social connectedness reported less frequent communication with their social networks, but expressed satisfaction with the amount of contact. For example, one caregiver with high connectedness had a long standing weekly scheduled call with a family member. Another caregiver did not communicate frequently with their social networks due time constraints; however, they described communication as appropriate to their needs.

By contrast, caregivers with low social connectedness lacked regular or frequent contact with their social networks. One caregiver indicated they wanted to interact more with others, although did not want to burden their friends. Another caregiver described frequent contact with one friend, also in a caregiving role, however, still reported feeling isolated from others.

### Supportive communication with people in the social network

Four subthemes emerged within the theme of supportive communication with people in the social networks: (a) open and honest conversations about cancer and demands of the caregiving role, (b) people regularly “checking in” to offer support, (c) validation and appraisal by those in their networks, and (d) empathy from other carers who have “walked that road”.

#### Open and honest conversations about cancer and the demands of the caregiving role

Talking openly and honestly with people about caregiving helped participants cope with the impact of cancer and caregiving demands. Most caregivers who reported open communication with others also reported moderate to high social connectedness scores. This open communication enabled some caregivers to make sense of, and to gain a different perspective on, their situation. One caregiver noted that, although it was emotionally challenging to speak openly about the cancer immediately following diagnosis, it became easier as the caregiver learned to accept the situation. Another caregiver felt it was easy to speak about their caregiving responsibilities because cancer became “part of their normal life”, and rather than an emotional unburdening; managing the cancer diagnosis and providing updates to people were “normalised”. Some caregivers described the benefit of talking to people outside of their family network, which alleviated the risk of burdening their family.

#### People regularly “checking-in” to offer support

Intertwined with having open and honest talks with those in their social networks, caregivers with moderate-high social connectedness described regular “check-ins” from family members, friends, and colleagues. Contact would be made via telephone, text message, or face-to-face, fostering opportunities for caregivers to talk openly about their lives. People who could be trusted to listen to the caregiver, were understanding and empathic, and made themselves available when caregivers needed to talk. These people were described as “close”, or within the caregiver’s inner social circle, and could be relied on for emotional support. Practical support would also be offered during these check-ins. They gave caregivers strength to “get through things, and cope with things” (CG05, female caring for partner). Talking with others also provided a welcome distraction for some caregivers, enabling them to take their mind away from their caregiving responsibilities.

#### Validation and appraisal from people in their networks

Individuals in the caregivers’ “close” networks were also validated the caregiver, through words of support and encouragement, which enabled caregivers to feel understood and seen. Caregivers also described being reassured by friends that their feelings and experiences were “appropriate”.

#### Understanding from other carers who have “walked that road”

Several caregivers described the importance of talking to people who had “walked that road” and could fully comprehend caring responsibilities, and thus understand and empathise with their situation. One caregiver, with low social connectedness, valued having a friend also in a caregiving role, who could understand their circumstances. This contrasted with their frustrations and perceived isolation when communicating with others in their social network, whom the caregiver described as not understanding their situation and responsibilities. Other caregivers described the usefulness of meeting and talking to other carers at hospitals, which put their own experience in context.

### Challenges and barriers when talking to others and seeking support

Caregivers identified several challenges when talking to, and seeking support from others, including (a) a reluctance to burden their family members and friends, (b) a belief that others do not understand their circumstances, and (c) a lack of trust.

#### Reluctance to burden family members and friends

Several caregivers with low social connectedness were reluctant to talk about cancer and caregiving with others, explaining that they needed to protect them from worry or distress, and minimise their burden. These caregivers believed family members and friends had their own responsibilities and problems to consider and that they would not want added stress. One caregiver, with moderate social connectedness, described protecting her adult children from additional care responsibilities, which limited how much information she disclosed.

Other caregivers with low connectedness believed disclosing their situation or emotions would be perceived as complaining, and that friends would not want to listen. As a result, caregivers reported not talking to others about their emotional state and their circumstances; it was “just easier to get on with it” (CG01, male caring for parent).

#### Belief that others do not understand their circumstances

Several caregivers described others as unable to understand or empathise with their caregiving and thus, they chose to remain silent. One caregiver described frustration when friends did not understand the impact of the caregiving, or minimised the burden (e.g., “*it can’t be that hard to do that*” [CG12, male caring for parent]), contributing to their feelings of isolation. Other caregivers described friends as uncomfortable during conversations about caregiving and cancer and uncertain about whether to raise the topic, which led to challenges openly talking about their circumstances.

#### Lack of trust

Receipt of support from others was also dependent on the caregivers’ trust in their social networks. Caregivers with low social connectedness found it difficult to trust others easily, or place trust in people whom they had known for a short time, and thus, would not disclose personal information. One caregiver believed they could only rely on themselves and needed to struggle through the caregiving situation on their own. Lack of trust and stoicism resulted in some caregivers being unable to talk openly about their caregiving role.

### Receiving instrumental support from social networks

Caregivers with moderate to high social connectedness described a network of family members and friends they could rely on for instrumental support, including help with transport and attending appointments with the care recipient. Family members and friends would also assist with errands, provide meals, and help with household tasks and entertaining the children. Notably, one caregiver with high social connectedness, who reported limited practical support received from family members due to their living overseas, described neighbours who provided substantial practical support in the form of driving the caregiver to/from public transport since the care recipient could no longer drive, as well as assisting the care recipient with groceries.

### Impact of the carer role on friendships and community activities

Several caregivers spoke of the impact of their caregiving on existing friendships related to (a) changes in the perceived value of social interactions; (b) lack of time to develop and maintain friendships, and (c) changes to friendships due to the cancer diagnosis.

#### Changes in the perceived value of social interactions

Several caregivers described changes to the perceived value of friendships in their social networks after the care recipient’s cancer diagnosis. One caregiver described socialising less with friends because they had more important priorities, such as providing care to their care recipient. Other caregivers reported less tolerance for the effort required in maintaining friendships, and less patience for others,“*[I] can’t be bothered going out to dinner with people and making small talk*” (CG15, female caring for partner).

#### Lack of time and capacity to develop and maintain friendships and community ties

Several caregivers with low to moderate social connectedness described a lack of time to develop and maintain friendship networks since providing care, despite feeling isolated. One caregiver with low social connectedness reported that caregiving prevented them from accepting impromptu social invitations, limiting their ability to socialise and adding to feelings of loneliness. A caregiver with moderate connectedness described the lack of mental capacity to invest in friendships while juggling caregiving and other responsibilities. Caregivers described not wanting to let others down in their social networks by agreeing to an activity, then cancelling. Several caregivers, with moderate connectedness, lamented the lack of time and capacity to participate in hobbies and community activities contributing to perceived isolation.

#### Changes to friendships due to the cancer diagnosis

Some caregivers with low connectedness spoke of unexpected changes to friendships following the care recipient’s cancer diagnosis. Some “all weather” friends became more reliable; other “fair-weather friends” were less faithful.

### Factors that fostered perceived belongingness

Caregivers were asked what helped them feel like they belonged within their community or social groups. Open communication and feeling understood and supported by others were closely intertwined with perceived belongingness within a community or social group. Feeling understood by others was an important bond developed over a long period of time and that it had an important role to play in their wellbeing. Friends in the caregivers’ networks who had similar “lived experiences” were able to express understanding and empathy and provide relevant support when needed. This perceived understanding, and acceptance by others, contributed to caregivers’ sense of belongingness within their social networks and communities. By contrast, one caregiver with low social connectedness indicated that “less than 10%” of people in their social networks understood their caregiving experiences.

The ability to provide mutual support and assistance to one another was considered another factor in fostering perceived belongingness. Offers of support that were open-ended and genuine enabled caregivers to feel they belonged. One caregiver described perceived belongingness within social groups as reflecting shared understanding of common goals, interests, and personal beliefs.

## Discussion

The current study sought to identify factors which contributed to perceived social connectedness in caregivers of people with cancer. Key themes emerged from the interviews captured the following: people in caregivers’ networks and communication frequency; supportive communication with people in the social network; challenges and barriers when talking to others and seeking support; receiving instrumental support from social networks; impact of the carer role on friendships and community activities, and factors that fostered belongingness.

Social connectedness is not determined by the number of connections or frequency of contact but by an internalised sense of connection. Those with moderate to high social connectedness scores tended to describe close relationships with family members, who provided both emotional and instrumental support. These caregivers also described frequent or regular communication with people in their social networks, which enabled them to feel understood, valued, and cared for. Meaningful relationships, characterised by mutual concern, trust, acceptance, and feeling valued have been shown to have positive impacts on health and wellbeing [19, 20]. These close relationships enable individuals to disclose emotional or personal issues, and to seek support and advice when needed [21]. In the current study, caregivers described these close, meaningful relationships as being developed over time, through reciprocal care, and/or through mutual interests. By contrast, several caregivers with low social connectedness described lack of trust in others, limiting their capacity to self-disclose, connect with others, and to feel supported. Self-disclosure is considered a key component in developing, maintaining, and fostering closeness and intimacy in social relationships [22]. However, self-disclosure also involves risk of negative evaluation and rejection; taking such a risk requires individuals to perceive the recipient as trustworthy, and that revealing one’s vulnerabilities will be received with warmth and understanding [23]. Social relationships develop through gradual disclosure of personal and intimate information about oneself [24]. Findings suggest that fostering beliefs and trust that the recipient will respond in a non-judgmental manner, in addition, skills to gradually disclose information about oneself to others, is likely reduce social isolation in caregivers.

The findings support existing literature that describes caregiving as an isolating and lonely experience [25, 26], with some caregivers describing others as unable to understand or empathise with their circumstances. Some caregivers did not want to burden others by talking about their caregiving experiences or leaning on them for emotional support, which contributed to their social isolation and loneliness. Meeting with, and talking to other caregivers, has been shown to enable caregivers to feel understood and less isolated [27]. In the absence of close family relationships and/or social networks, the findings suggest that opportunities to meet with other caregivers, for example, through access to caregiver support groups [28], may be particularly beneficial by allowing caregivers to share their experiences with others who understand.

The current study is not without limitation. Although we sought to equally involve male caregivers, most participants were female. Minimal information on demographic characteristics was collected in order to minimise participant burden, which limits understanding of participant factors that may influence social connection, such as education level, employment status, cultural and linguistic diversity, and/or number of dependent children. In addition, open-ended questions were used to ascertain the size of caregivers’ social networks, which limits accurate understanding of network size. To build on our findings, further research should consider oversampling male caregivers, a quantitative assessment of network size, and further understanding of demographic characteristics which may influence social connection.

Despite these challenges, our findings have captured insights into the key role of social networks on emotional support for cancer caregivers, and how support strategies can be developed for caregivers who are isolated and lack connection with others. Further research that builds on these insights and identifies strategies to promote social connection for those who lack strong supportive networks is warranted. Pilot research from the USA has shown promise of a positive activity intervention program on improving perceived social connectedness in a sample of people with clinical depression or anxiety [29]. There is the potential to examine how such activities could be implemented with caregiver samples to enhance social networks and perceived connectedness, and subsequently, wellbeing in these populations.

## Conclusion

Social connectedness is increasingly recognised as influencing health outcomes across populations. For caregivers of people with cancer, our qualitative findings suggest the importance of receiving emotional and instrumental support from social networks to cope with, and alleviate the stress and strain of providing care. For caregivers with low social connectedness, research is needed to develop and evaluate strategies and interventions to enhance perceived connections, and ultimately improve health and wellbeing.

## Data Availability

Data will be provided upon reasonable request from authors.
